# A comprehensive dataset of near infrared spectroscopy measurements to predict nitrogen and carbon contents in a wide range of tissues from *Brassica napus* plants grown under contrasted environments

**DOI:** 10.1016/j.dib.2024.111163

**Published:** 2024-11-23

**Authors:** Sophie Rolland, Françoise Leprince, Solenn Guichard, Françoise Le Cahérec, Anne Laperche, Nathalie Nesi

**Affiliations:** IGEPP, INRAE, Institut Agro, Université de Rennes, Le Rheu 35650, France

**Keywords:** *Brassica napus*, Dataset, Near infrared spectroscopy, N and C contents, Abiotic stress, Calibration model, Prediction

## Abstract

Winter oilseed rape (WOSR, *Brassica napus* L.) is the third largest oil crop worldwide that also provides a source of high quality plant-based proteins. Nitrogen (N) and carbon (C) play a key role in plant growth. Determination of N and C contents of plant tissues throughout the growth cycle is crucial in assessing plant nutritional status and allowing precise input management. In the dataset presented in this article, 2427 WOSR samples arising from a large diversity of tissues collected on WOSR diversity were analyzed by near infrared spectroscopy from 4000 to 12,000 cm^−1^. At the same time, reference chemical data for the N and C contents of the same samples were determined by elemental analysis using the Dumas method. Partial least squares regression has been used to develop predictive models linking spectral and chemical data, so that new samples can be characterized without the need for reference methods. This dataset could be used to test new calculation algorithms in order to enhance prediction performance or for training purposes. These models can be used as a rapid method for determining N and/or C content, adding to decision-support tools for fertilizer application throughout the plant developmental cycle.

Specifications Table

**Table:** 

Subject	Chemistry: Analytical Chemistry: Spectroscopy Agricultural and Biological Sciences
Specific subject area	Spectral and chemical characterization of winter oilseed rape (WOSR) tissues
Type of data	Table, Figure, Spectroscopic data. Raw data presented as .csv file formats.
Data collection	Tissues were collected on winter oilseed rape accessions grown under contrasted environmental conditions. All data were all recorded in 2017 on samples of homogeneous ground dried tissues. Spectral data were acquired using a Fourier transform near infrared spectrometer (MPA, Multi Purpose FT-NIR Analyser – Bruker Optics GmbH, Ettlingen, Germany). Each spectrum was collected in reflectance mode covering wavenumbers from 4000 to 12,000 cm^−1^ with a 16 cm^−1^ optical resolution and resulted from an average of 64 successive data acquisitions. Spectral data were expressed in absorbance per each wavenumber (in cm^−1^). N and C contents were determined by the Dumas combustion method using an automated CN analyzer (Elementar Vario Micro cube CHNS – Elementar Analysensysteme GmbH, Germany). N and C contents were expressed in % of dry matter.
Data source location	Institution: Institute of Genetics, Environment and Plant Protection (IGEPP); INRAE, Institut Agro, University of Rennes City/Town/Region: 35,650 Le Rheu Country: France
Data accessibility	Repository name: Data INRAE (https://data.inrae.fr/) Data identification number: 10.57745/6VYUQN Direct URL to data: https://entrepot.recherche.data.gouv.fr/dataset.xhtml?persistentId=doi:10.57745/6VYUQN
Related research article	None

## 1 Value of the Data

• The dataset establishes a link between spectral properties and chemical composition (N, C) of a wide variety of plant tissues in winter oilseed rape. The prediction models can be used by diverse communities (scientists, breeders, producers).
• NIR spectra data and calibrations can be re-modelled by adding new spectra and running regression algorithms to improve prediction performances.
• Calibration models database can be imported and transferred onto other near infrared spectroscopy (NIRS) instruments.
• The present dataset can be used to test new chemometric methods and also for training.

## 2 Background

The goal of the present dataset was to gather near infrared spectral data with reference chemical data from a large population of winter oilseed rape plant samples in order to define robust NIRS-based predictive models to estimate the contents in N or C in additional samples.

## 3 Data Description

**Fig. 1:**
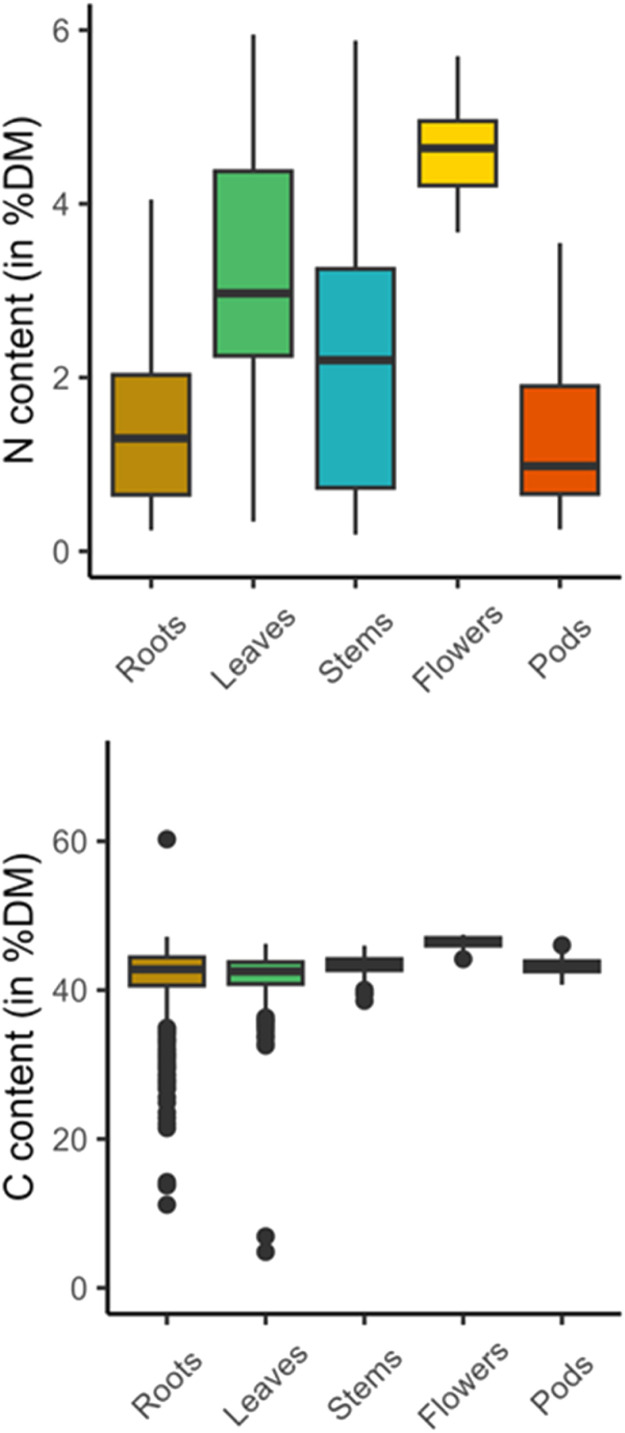
Distribution of N and C contents (% of dry matter) within the whole sample collection (*n*= 2427).

**Fig. 2:**
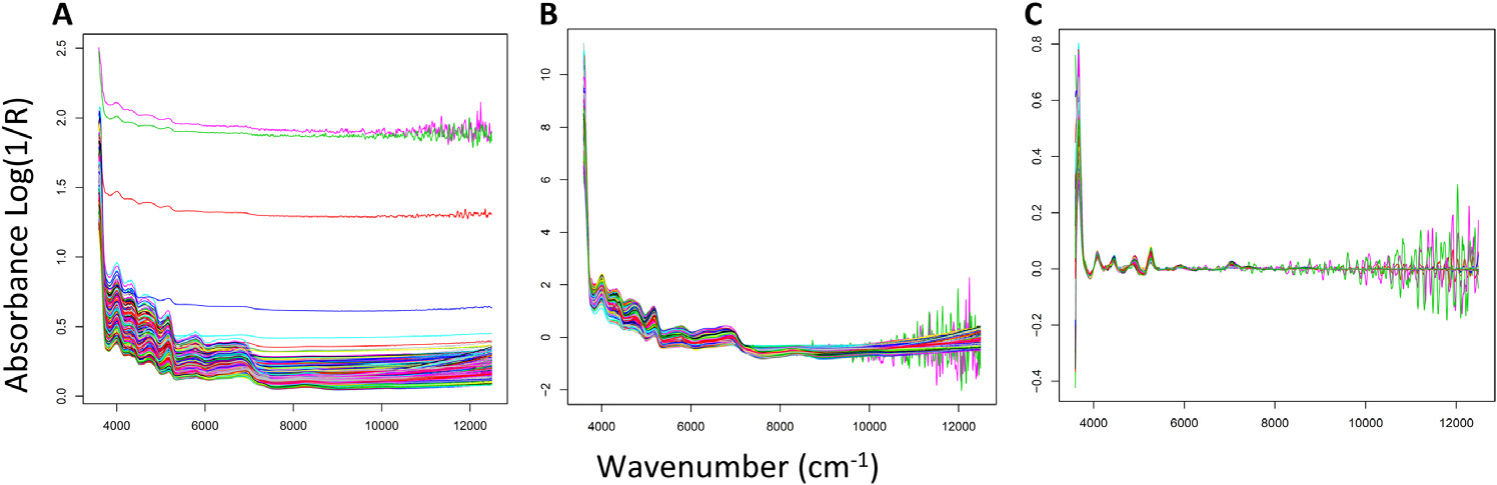
Near infrared absorbance spectra of 2427 oilseed rape samples. Spectra were acquired on wavenumbers from 4000 to 12,000 cm^−1^ and shown before pretreatment (**A**), after a pretreatment using standard normal variate (SNV) (designated by the OPUS8.1 software as the optimal pre-treatment for the N predictive model) (**B**), and after a combined pretreatment using SNV and first derivate (designated by the OPUS8.1 software as the optimal pre-treatment for the C predictive model) (**C**).

**Fig. 3:**
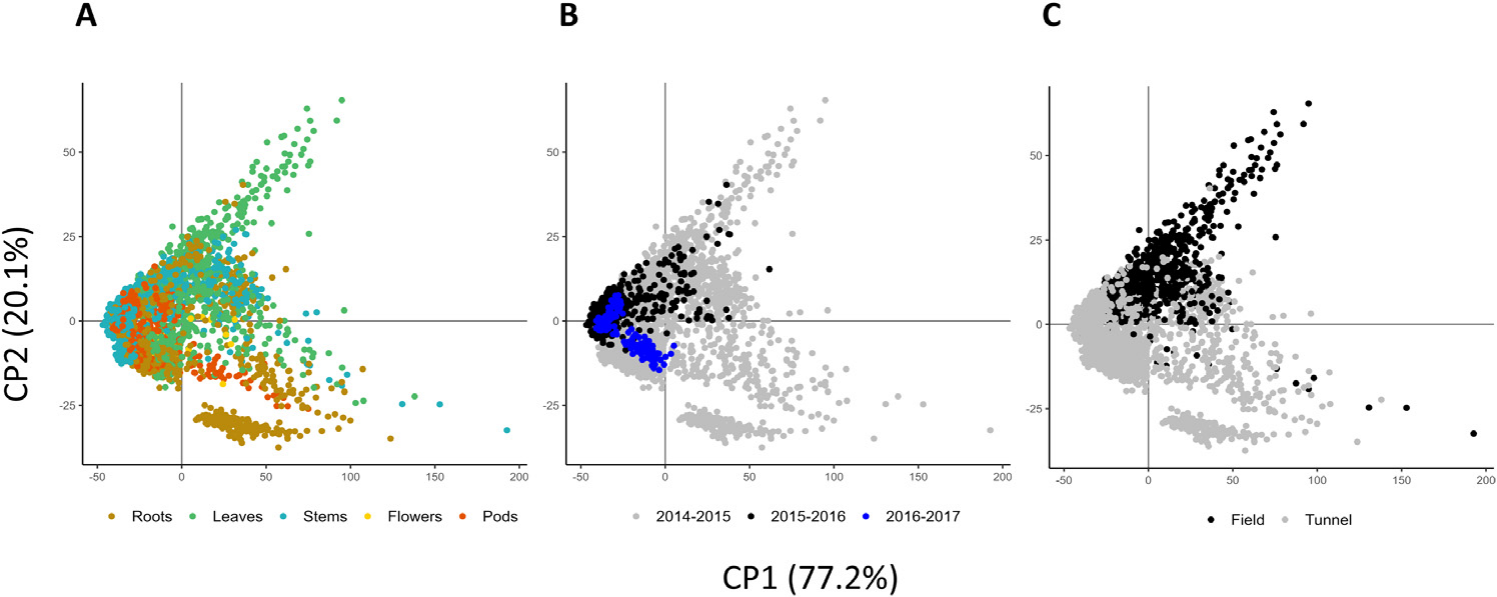
Projection of the first two principal components (CP1 and CP2) of a PCA of raw spectral data after removing the outliers. The scatterplot has been colored according to tissue (**A**), crop season (**B**) or growing conditions (**C**).

**Fig. 4:**
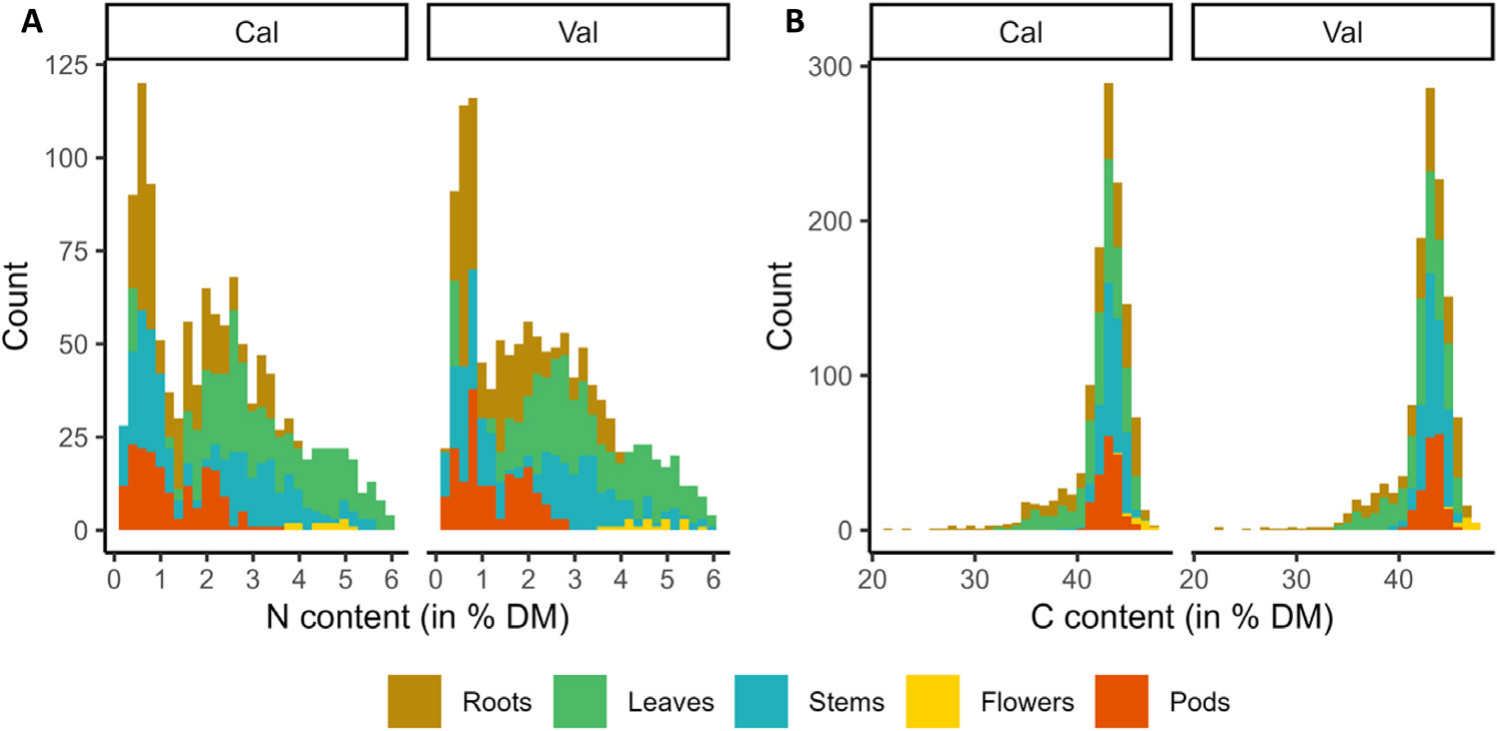
Histogram of N content (A) and C content (B) in the samples used for model calibration (Cal) or validation (Val) after removing the outliers.

**Fig. 5:**
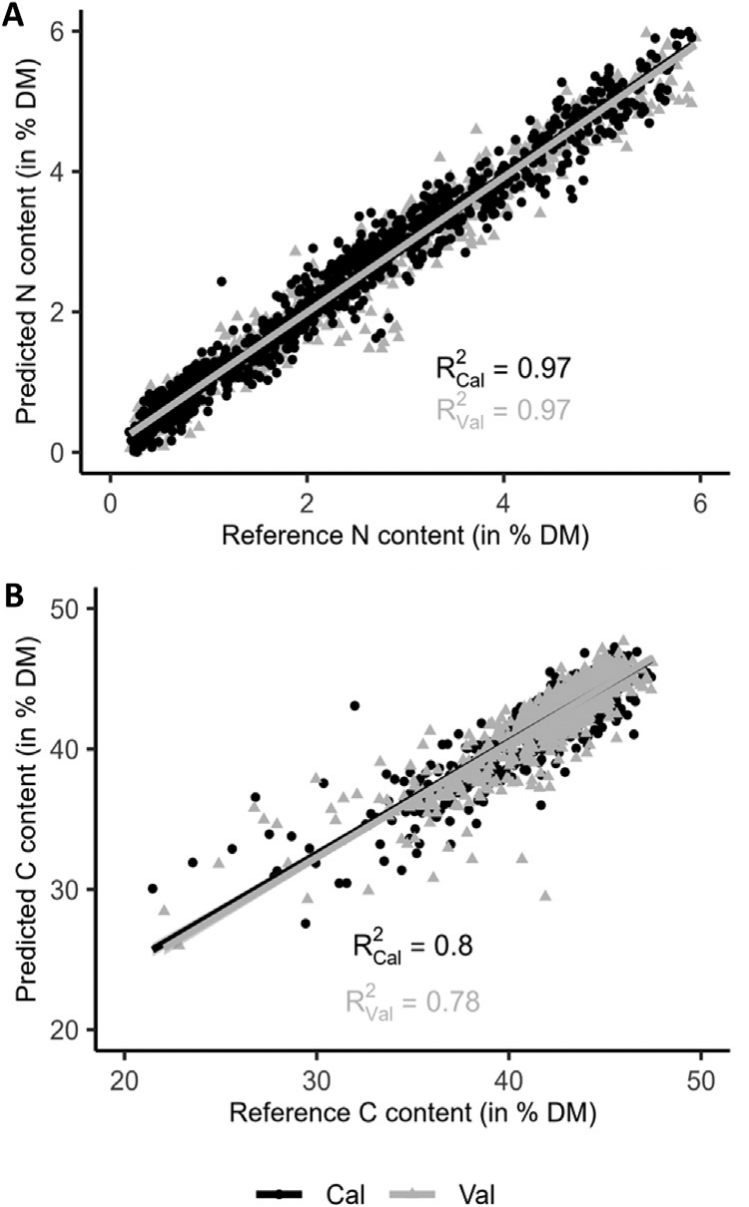
Prediction models for N content (A) or C content (B). Data were depicted regarding calibration (Cal, black circles) or validation (Val, grey circles) datasets. The linear regression curve between measured reference values and the values predicted from near infrared spectra are given with the corresponding coefficients of determination (R²) for each dataset.

The ambition of the present study was to set up precise and predictive NIRS models to estimate the N and C contents in tissues of WOSR. As a consequence, a first effort was to collect the most representative sample set as possible. For that purpose, we gathered a total of 2427 samples of WOSR plants maximising genetic and ecological diversity (Table 1). N and C contents were scored in each sample through elementary analyses (Table 2, Fig. 1). In parallel, near infrared spectra data of the same samples were measured and collected at absorbance spectrum in the presence of energies in wavenumbers 4000–12,000 cm^−1^ (Fig. 2). Outliers were detected by the software OPUS8.1 and removed before calculation of the predictive models. These outliers concerned 15 and 8 samples regarding N and C measures respectively (Tables 2, and 3). A principal component analysis (PCA) of the whole spectra dataset showed that 97.3 % of the sample set diversity was captured by the two first axes (Fig. 3). In addition, no specific pattern was observed indicating that the spectra can be considered as a single population, and a unique calibration can be done, even though samples originated from multiple genotypes, growing conditions and tissues. The whole sample dataset was then equally divided into two sets, one for the calibration (CAL) and the other one for the validation (VAL) that displayed equivalent sample number and N/C distribution (Table 2, Fig. 4). Partial Least Squares (PLS) predictive models for N and C contents were developed using the OPUS8.1 software with the statistics presented in Table 3. The predictive equation for N content was developed with 17 PLS factors on the spectral range from 4242.9 to 7506.1 cm^−1^. The standard error in calibration or prediction are respectively 0.244 (RMSEC) and 0.248 (RMSEP). A very high accuracy between NIRS predicted values and the reference values was obtained for both calibration and validation sample sets (R² = 0.97; Fig. 5). The ratio of performance to deviation (RPD) is high: RPD = 5.93. The predictive equation for C content was developed with 7 PLS factors on the spectral range from 6094.4 to 9403.8 cm^−1^. The RMSEC and RMSEP were of 1.31 and 1.32 respectively. The accuracy between predicted and reference C values was lower than those obtained with N values (R²cal = 0.8 and R²val = 0.78; Fig. 5). The RPD was of 2.12.

**Table 1:** Main characteristics of the whole set of samples.

	Growth condition	Field				Tunnel						TOTAL (*n*= 2427)
	Growth season	2014–2015		2015–2016		2014–2015		2015–2016		2016–2017		
	Nutrition regime^a^	N-	*N*+	N-	*N*+	N-	*N*+	W-	*W*+	W-	*W*+	
**Tissues**	**Roots**	13	13	10	12	228	238	48	36	30	10	**638**
	**Leaves**	222	236	16	18	115	124	0	0	30	10	**771**
	**Stems**	142	137	11	12	92	111	48	36	30	10	**629**
	**Flowers**	0	0	0	0	11	21	0	0	0	0	**32**
	**Pods**	0	0	0	0	133	148	24	12	30	10	**357**

a Plants were grown under optimal (+) or suboptimal (-) conditions of nitrogen nutrition (N) or watering (W) as described in the experimental design.

**Table 2:** Summary statistics of N and C contents of WOSR samples used for prediction models.

Chemical analyses (% dry matter)	n	Min.	Max.	Mean	SD
N content in total sample set	2427	0.19	5.95	2.20	1.46
N content in calibration sample set (CAL)^b^	1205	0.19	5.95	2.21	1.47
N content in validation sample set (VAL)^c^	1207	0.2	5.95	2.19	1.47

C content in total sample set	2427	4.83*	60.26^a^	42.41	3.28
C content in calibration sample set (CAL)^b^	1210	21.46	47.41	42.43	2.9
C content in validation sample set (VAL)^c^	1209	22.06	47.46	42.53	2.8

a outlier samples (outliers were excluded in the CAL and VAL datasets).

b CAL, calibration set.

c VAL, validation set.

**Table 3:** Prediction perfomances of the N and C models.

	n	n outliers	n CAL (n fix / n rotating)	n VAL (n fix / n rotating)	PLS-R factors	Spectra pretreatment	Concentration range (%DM)	Spectral range (wave numbers in cm^−1^)	RMSEC	RMSEP	RPD	R²
**N model**	2427	15	1205 (1116/89)	1207 (1114/93)	17	SNV	0.2–5.9	4242.9–7506.1	0.244	0.248	5.93	0.97
**C model**	2427	8	1210 (1118/92)	1209 (1110/99)	7	SNV and first derivate	21.5–47.5	6094.4–9403.8	1.31	1.32	2.12	0.78

n, number of samples; CAL, calibration; VAL, validation; fix, fixed glass cup of 20 mm used for NIR spectra acquisition; rotation, rotative quartz cup of 51 mm ∅ used for NIR spectra acquisition; PLS-R, partial least squares regression; RMSEC : root mean square error in calibration; RMSEP : root mean square error in prediction; RPD : ratio of performance deviation; R² : coefficient of determination.

## 4 Experimental Design, Materials and Methods

### 4.1 Plant growth conditions and sampling

Plant accessions were chosen to span the genetic diversity in winter oilseed rape (WOSR) regarding the registration year (1965–2011) and type with 6 varieties rich in erucic acid (C22:1) and glucosinolates (GSL) (WOSR_++), 7 poor in C22:1 and rich in GSL (WOSR_0+) and 49 poor in C22:1 and in GSL (WOSR_00). Samples from roots, leaves, stems, flowers or pods were collected from WOSR plants grown under a wide range of environmental conditions: three growing seasons (2014–2015; 2015–2016; 2016–2017); two growth conditions (fields or tunnels); two N nutrition regimes as described by [1] (*N*^+^, optimal N supply for a target seed yield of 3.5 tha^−1^; *N*^−^, suboptimal N regime corresponding to the *N*^+^ supply lowered by 80–100 kg.ha^−1^ of N); two watering levels as described by [2] (*W*^+^, optimal watering for which the water potential of the substrate was maintained above -200 mbar throughout the growth cycle; *W*^−^, suboptimal watering for which the water potential of the substrate was lowered and maintained around -600 mbar). Samples were collected on plants at 5 developmental stages as described by [3] : 15–19 stage corresponding to the rosette development (5 to 9 first leaves); 31–35 stage corresponding to the bolting stage (1 to 5 internodes); 55–59 stage corresponding to the emergence of the main inflorescence (individualised closed buds); 61–65 stage corresponding to flowering (10–50 % open flowers on the main inflorescence); 70–75 stage corresponding to seed development (10–50 % of the pods reach their final size); 79–85 stage corresponding to seed maturation (10–50 % of the pods reach full maturity) and 89–91 stage corresponding to full mature senescent plants (ca. 950 growing degree days after flowering). Samples were oven-dried at 80 °C for 48 h and grinded with the TissueLyser II system (Qiagen, Hilden, Germany).

### 4.2 Nitrogen and carbon content

C and N contents were determined by the Dumas combustion method [4] using an automated CN analyzer (Elementar Vario Micro cube CHNS; Elementar Analysensysteme GmbH, Germany). 5–10 mg of dry ground samples were used (precision weighing at 0.001 mg) with a supplemental drying phase of 12 h at 80 °C before analyses. Two standard samples were included in each series of analyses to ensure accuracy of the N and C concentration measurements (URSAVE, V464 oak leaf (N%: 0.944 ± 0.02, C%: 49.2 ± 0.3) and V463 maize (N%: 1.28 ± 0.03)). N and C contents were expressed in % of dry matter. In parallel, an estimate of the measurement error on the reference data by the chemistry reference Dumas combustion method was carried out. The average deviation for the 10 measurements on 6 samples chosen to have various concentrations (1.7 % N to 4 % N) is 0.066 (data not shown).

### 4.3 NIR spectra acquisition

Near infrared spectra acquisition was performed on ∼ 5 g of homogeneous ground dried tissue at room temperature using a completely automated Fourier transform near infrared (FT-NIR) spectrometer (MPA, Multi Purpose FT-NIR Analyser; Bruker Optics GmbH, Ettlingen, Germany). Spectral data were recorded as absorbance data in the presence of energies in wavenumbers 4000–12,000 cm^−1^ with a spectral resolution of 16 cm^−1^. Dry ground samples were transferred into a rotative quartz cup of 51 mm diameter (called “rotating”) or a fixed glass cup of 20 mm diameter (called “fix”) according to the quantity of powder available (Table 3). Each spectrum resulted from an average of 64 co-added scans. To reduce possible device drift, a background spectrum measurement was performed every four hours during series.

### 4.4 Predictive models

The N and C predictive models have been developed independently but using the same workflow. First, PCA was performed on R software on all the spectra in order to verify a continuum in our data. After removing outliers, the data were randomly subdivided (50/50) into a set of calibration (CAL) and a set of test representative of the variability (VAL) of the data. The optimal pretreatment of NIR spectra was determined [5]. A standard normal variate (SNV) approach was designated as the optimal pre-treatment for the N predictive model (Fig. 2B) and a combined pretreatment using SNV and first derivate was designated as the optimal pre-treatment for the C predictive model (Fig. 2C). Prediction models were performed using Partial Least Squares (PLS) regression on, with first the elimination of non-informative part of the spectra and secondly the determination of the optimal number of factors to explain the model (Table 3). Model performances was estimated on the basis of the following statistical parameters: coefficient of determination (R²), root mean square error of calibration (RMSEC) calculated on the calibration dataset, root mean square error of prediction (RMSEP) calculated on the validation dataset, ratio of performance to deviation (RPD) calculated as the standard deviation divided by the standard error of prediction. All these steps have been optimized using the OPUS software v 8.0.19 (Bruker Optics GmbH, 2017).

## Limitations

Not applicable.

## Ethics Statement

The current work does not involve human subjects, animal experiments, or any data collected from social media platforms.

## CRediT authorship contribution statement

**Sophie Rolland:** Investigation, Software, Data curation, Visualization, Writing – original draft. **Françoise Leprince:** Investigation. **Solenn Guichard:** Investigation. **Françoise Le Cahérec:** Conceptualization, Methodology, Writing – review & editing. **Anne Laperche:** Supervision, Conceptualization, Methodology, Writing – review & editing. **Nathalie Nesi:** Supervision, Conceptualization, Methodology, Writing – original draft, Writing – review & editing.

## Data Availability

DataverseFull_dataset (Original data)
